# Timing of exercise differentially modulates fear memory and hippocampal neurotransmitters in male rats

**DOI:** 10.3389/fnins.2026.1824029

**Published:** 2026-05-01

**Authors:** Samuel Bennett, R. Shelby Blair, Kaushik Avadhanula, Mitchell H. Birkett, Citlali Palapa, Stephen Maren, Naomi Nagaya, Shogo Sato

**Affiliations:** 1 Department of Biology, Center for Biological Clocks Research, Texas A&M University, College Station, TX, United States; 2 Department of Psychological and Brain Sciences, Texas A&M University, College Station, TX, United States

**Keywords:** circadian rhythms, exercise, exercise timing, context fear, hippocampus, neurotransmitter, rat

## Abstract

Exercise promotes neurogenesis and enhances memory consolidation while reducing the retention of aversive memories and anxiety-like behaviors. While our previous work found that acute exercise alters neurotransmitter concentrations, including dopamine and serotonin, in a time-of-day-dependent manner, the long-term effects of chronically timed exercise on neurotransmitter dynamics and behavioral phenotypes remain unclear. To examine whether the daily timing of a chronic exercise intervention modulates its impact on neurotransmitter profiles and fear responses, male rats were conditioned using a Pavlovian contextual fear approach, then assigned to a 4-week treadmill exercise intervention performed during the early (ZT14) or late (ZT22) active phase or a time-matched sham-exercise control group. One day after completing training, rats underwent a context retrieval test in the middle of active phase (ZT18), and hippocampal neurotransmitters were quantified using UPLC–MRM/MS. Rats subjected to sham-exercise at ZT22 exhibited higher freezing than sham-exercised rats at ZT14, whereas exercise interventions at ZT22 selectively attenuated freezing. Histamine, acetylcholine, and GABA exhibited significant exercise × time interactions. Direct neurotransmitter–freezing correlations were weak after false discovery rate control, consistent with a network-level reorganization rather than a single transmitter driver. These findings suggest that vulnerability to aversive memory expression can be buffered by exercise, if timed appropriately, and that exercise reshapes hippocampal neuromodulatory tone in a circadian–phase–dependent manner, supporting the potential of exercise timing as a chronotherapeutic strategy to enhance stress resilience and mental wellbeing.

## Introduction

The circadian clock, a biologically conserved system, induces daily rhythms of physiological functions, including sleep-wake cycles, metabolism, hormone release, and mood (Bass and Takahashi, 2010; Green et al., 2008; Panda, 2016). The central clock, located in the hypothalamic suprachiasmatic nucleus (SCN), is entrained by light as an external timed cue (zeitgeber), thereby synchronizing peripheral clock oscillations and governing systematic circadian homeostasis. Independently of light, non-photic zeitgebers such as food intake and physical activity can also entrain peripheral clock rhythmicity. The circadian system enables organisms to temporally control and anticipate biological processes in preparation for food intake and physical activity (Asher and Sassone-Corsi, 2015; Gabriel and Zierath, 2019; Longo and Panda, 2016; Manoogian and Panda, 2017) and is crucial for maintaining organismal homeostasis and health (Bennett and Sato, 2023). Thus, the circadian clock imposes temporal boundaries on physiological functions and behaviors. For instance, time of day optimizes physiological responses to physical exercise (Akasaki et al., 2006; Kemler et al., 2020; Sato et al., 2019, 2022; Savikj et al., 2019, 2022) and food intake (Acosta-Rodríguez et al., 2022; Deota et al., 2023; Greenwell et al., 2019; Hatori et al., 2012).

Mental health disorders affect one in three people globally (Vigo et al., 2016), with growing evidence that exercise improves mental wellbeing via improved mood, lowering stress, and increasing hippocampal neurogenesis (Anderson and Shivakumar, 2013; Bartholomew et al., 2005; Broman-Fulks et al., 2004; Byrne and Byrne, 1993; DeBoer et al., 2012). Neurogenesis is dramatically increased by exercise and has anxiolytic effects in animal models (Scott et al., 2021). Exercise-induced modulation of hippocampal neurotransmitters such as dopamine, serotonin, and norepinephrine may also regulate fear and anxiety (Maren and Holmes, 2016). These neurotransmitters are synthesized through amino acid metabolism and maintain psychiatric homeostasis. Notably, our previous study revealed that some neurotransmitters, including dopamine and serotonin and their catabolites are only increased in response to acute exercise at the early active phase [Zeitgeber time (ZT) 15] but not following exercise at the early rest phase (ZT3) (Sato et al., 2022). Thus, in addition to the quantity, duration, and type of exercise, the time of day that exercise is performed may impact psychiatric outcomes, although the effects of chronically timed exercise intervention on neurotransmitter dynamics and behavioral phenotypes remain unexplored.

To investigate this, we employed a hippocampal-dependent contextual fear conditioning model to determine whether the timing of chronic exercise intervention differentially affects fear responses and hippocampal neurotransmitter dynamics in rats. Ultimately, we aim to provide novel insights into how timed exercise interventions can be leveraged to improve mental health.

## Materials and methods

**FIGURE 1:**
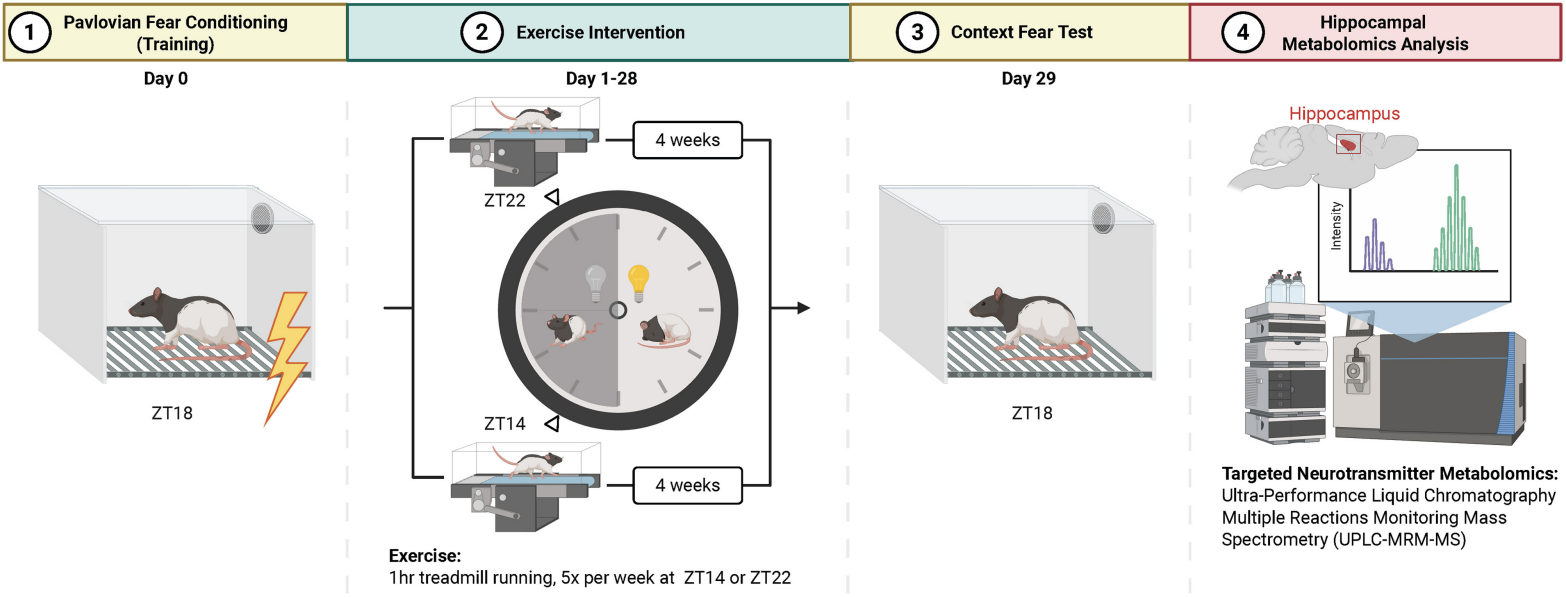
Schematic overview of experimental workflow. (1) Rats were conditioned to aversive context stimulus during the middle of the active phase (ZT18); (2) followed by a 4-week, differentially timed exercise intervention where all sessions were completed at ZT14 or ZT22; (3) 1 day after the end of exercise intervention, rats completed a follow-up context fear test at ZT18 followed by hippocampal dissection; (4) hippocampal neurotransmitter quantification by mass spectrometry. Figure created with Biorender.com.

In brief, adult male Long-Evans rats were housed under 12:12 h reverse light-dark (LD) conditions (room temperature at 23 °C ± 1 °C, relative humidity at 40%–60%) with a standard chow diet (Teklad 8604) and water available *ad libitum*. Rats underwent a standard fear conditioning procedure, which results in aversive memories that induce freezing behavior before completing a 4-week timed exercise intervention. Animals were assigned to experimental groups using simple randomization by drawing randomly assigned numbers, without stratification or blocking (*N* = 6 per group): exercise or sham-exercise in the early active phase (zeitgeber time (ZT) 14) or late active phase (ZT22). After the exercise training intervention, rats underwent a context fear test before being sacrificed, and hippocampi were extracted for neurotransmitter quantification by mass spectrometry (Figure 1).

### Animals

Twenty-four adult male Long-Evans rats (200–224 g upon arrival) were purchased from Envigo (Indianapolis, IN) and individually housed in clear plastic cages on a 12:12 h reverse LD cycle [lights on at ZT0 (6:00 PM); lights off at ZT12 (6:00 AM)]. Animals were fed a standard lab chow diet and water *ad libitum*. Rats were habituated to experimenters via handling for ∼30 s per day for five consecutive days following arrival. All experiments were carried out per guidelines approved by the Texas A&M University Institutional Animal Care and Use Committee (AUP 2021-0110) and following policies of the National Institutes of Health Guides for the Care and Use of Laboratory Animals. In accordance with established guidelines (Garrigos et al., 2021) all methodological details and experimental design were thoroughly described to ensure reproducibility.

### Behavioral apparatus

All behavioral procedures occurred in eight identical observation chambers (30 × 24 × 21 cm; Med Associates, St. Albans, VT) composed of aluminum sidewalls and Plexiglas ceiling, rear wall, and front door. One sidewall contained an incandescent house light. Chamber floors consisted of 19 stainless steel rods (4 mm in diameter) spaced 1.5 cm apart (center to center) for foot-shock unconditioned stimulus (US) delivery. The rods were wired to a shock source and a solid-state grid scrambler (Med Associates). Beneath the rods was a removable tray for scent. In addition, each chamber was positioned in a sound-attenuating cabinet equipped with a ventilation fan to provide background noise (65 dB). All behavior procedures were conducted at the same time of day (ZT18), under dim red illumination (chamber house light fitted with a red filter; room lighting red), with no white light used during behavioral sessions, 1.5% acetic acid for scent, and open cabinet doors. Additionally, rats were individually transported to and from the vivarium in plastic boxes with light protection.

Locomotor activity was measured by recording the displacement of the load cell platform underneath each chamber (Maren, 1998). Before the experiment, each load cell amplifier was calibrated to a fixed chamber displacement, and the output of each amplifier was set to a specific gain to detect immobility. The output of the load cell amplifier was digitized such that one observation every 200 ms for each rat was recorded via the Threshold Activity software (Med Associates). Freezing behavior was derived from the locomotor activity and was quantified by computing the number of observations for each rat that had a value less than the freezing threshold as previously described (Maren, 1998). Behavioral assessments were automated and conducted entirely via the software.

### Behavioral procedure – fear conditioning

On the day immediately preceding the start of the exercise intervention (described below), rats individually underwent contextual fear conditioning in conditioning chambers (context A) at ZT18. After a 3-min baseline period, rats received five unsignaled foot shocks (unconditioned stimulus; 1 mA, 2 s duration) separated by 1-min inter-trial intervals (ITI). Following the final shock, rats remained in the chamber for an additional 1 min, after which freezing behavior was reassessed (post-training).

### Exercise protocol

Depending on group allocation, rats were subjected to either a 4-week treadmill (Columbus Instruments, Columbus, OH) running exercise intervention or a 4-week sham-treadmill intervention. Crucially, rats allocated to the sham-exercise group were treated identically to their exercise counterparts, with the exception that the treadmill they were placed in was non-operational (sham). Before the main exercise intervention, a 3-day acclimatization protocol (Sato et al., 2019; Treebak et al., 2014) was completed during the first week of exercise. On day 1, animals were placed in a stationary or sham treadmill, depending on the experimental group, for 5 min at their respective time points (ZT14 vs. ZT22) before completing 10 min of exercise (starting at 6 m⋅min^–1^ and increasing by 2 m⋅min^–1^ every 2 min until 12 m⋅min^–1^ at 5% gradient). The following day, rats completed a 15-min exercise bout with no rest period beforehand. Day 3 was a rest day (i.e., rats were left in cages and undisturbed) before the commencement of the exercise intervention proper. For the remainder of the week (Days 4–6), rats were exercised for 1 h (starting at 6 m⋅min^–1^ and increasing by 2 m⋅min^–1^ every 2 min until 12 m⋅min^–1^ at 5% gradient) with a rest day on day 7. Throughout the exercise intervention, rats completed exercise 5 days per week (days 1, 2, 4, 5, and 6). All bouts were 1 h long and commenced at 6 m⋅min^–1^, increasing by 2 m⋅min^–1^ every 2 min until 14 m⋅min^–1^, 16 m⋅min^–1^, 18 m⋅min^–1^, and 20 m⋅min^–1^ on weeks 2, 3, and 4, respectively.

### Context fear test

To evaluate the persistence or modulation of fear memory following the chronic exercise intervention, a test was conducted 1 day after the final exercise session at ZT18. Rats were re-exposed to the conditioning chamber for a 10-min context test under identical conditions as the initial conditioning session, with no shock administered. Freezing behavior, defined as the absence of all movement except respiration, was recorded and analyzed to assess the impact of exercise timing on fear memory retention.

### Hippocampal isolation

Immediately after the contextual fear retrieval test performed at ZT18, rats were euthanized by CO_2_ inhalation. The entire hippocampus was rapidly dissected from each rat between ZT18 and ZT19, flash-frozen in liquid nitrogen, and stored at -80°C until analysis.

### Neurotransmitter quantification

Targeted neurotransmitter metabolomics analyses were outsourced to Creative Proteomics (Shirley, NY) and were conducted by investigators blinded to group allocation. The mean Coefficient of variation for all metabolites was 2.8% ± 1.8%.

Amino acid type compounds (neurotransmitters): An internal standard (IS) solution containing 10 isotopically labeled internal standards for each analyte was prepared in 70% acetonitrile. Internal Standard solutions were then serially diluted 10 times for each compound. Hippocampi underwent bead-homogenization (2 beads, 3 × 1 min at 30 Hz) in IS solution at 10 μl per mg tissues for 1 min. Samples were then vortexed and sonicated for 30 s before centrifugal clarification. Fifty microliters of clear supernatant of each sample or standard solution were mixed with 100 μl of 20 mM dansyl chloride solution and 100 μl of borate buffer. The mixtures were incubated at 40 °C for 30 min. After the reaction, 5 μl of each solution was injected to run ultra-performance liquid chromatography multiple reactions monitoring mass spectrometry (UPLC MRM/MS) on a Waters ACQUITY UPLC system coupled to a Sciex QTRAP 6500 Plus mass spectrometer with positive-ion detection. LC separation was carried out on a C18 UPLC column (2.1 × 100 mm, 1.8 μm) with 0.1% formic acid in water and acetonitrile for binary solvent for gradient elution.

Corticosterone: Twenty microliters of the clear supernatant of each sample in the above was diluted 3-fold with water. In addition, serially diluted standard solutions of corticosterone were prepared in a solvent and a concentration-matched internal standard solution. Ten microliter aliquots of the standard and sample solutions were injected to run UPLC-MRM/MS on a Waters ACQUITY UPLC system coupled to a Sciex 7500 QQQ mass spectrometer with positive-ion detection. LC separation was carried out on a C18 UPLC column (2.1 × 150 mm, 1.8 μm) with 0.1% formic acid in water and 0.1% formic acid in acetonitrile for binary solvent gradient elution.

Calculation: Concentrations of the detected compounds were calculated with internal standard calibration by interpolating the constructed linear regression curves of individual compounds with the analyte-to-internal standard peak ratios measured from each sample solution.

### Data analysis

All data are reported as mean ± standard error. A statistical significance of *P* < 0.05 was used for all tests.

Fear conditioning: For training, the percentage of freezing was averaged across the 3-min baseline (BL) period, the 1-min ITI, and the 1-min post-trial period. During the context test, freezing was averaged every 60 s and across the total test for a 10 min mean. Due to the exclusion of one unhealthy animal, 1-min ITI context test data were analyzed by fitting a mixed model using a compound symmetry covariance matrix and fitted using Restricted Maximum Likelihood (REML) (GraphPad Prism 8.0). Between groups, context test data were compared using a two-way analysis of variance (ANOVA) with main effects of exercise condition (exercise vs. sham) and time of day (ZT14 vs. ZT22). Tukey’s *post hoc* analysis was used to identify significant pairwise comparisons between experimental groups for both statistical approaches.

Neurotransmitter metabolomics: Neurotransmitter concentrations are reported relative to hippocampal mass (nmol⋅g). Between groups, neurotransmitter concentrations were compared by two-way ANOVA with main effects of exercise condition (exercise vs. sham) and time-of-day (ZT14 vs. ZT22). To account for multiple testing across neurotransmitters, *P*-values for main effects and interactions were adjusted using the Benjamini-Hochberg false discovery rate (FDR) method. Both unadjusted (*p*) and adjusted (*q*) values are reported. Tukey’s *post hoc* analysis was used to identify significant pairwise comparisons between experimental groups. Where significant main effects were present, effect sizes were calculated using partial eta squared (η^2^p) with values of 0.01, 0.06, and over 0.14 considered small, medium, and large, respectively (Cohen, 1988). Pearson’s r^2^ correlation coefficient was calculated between variables to assess inter-neurotransmitter and behavioral data. To account for multiple testing, *p*-values from correlation analyses were adjusted using the FDR method. Data were presented as a series of correlation matrices, including all groups and individual matrices for each group.

## Results

### Fear conditioning

**FIGURE 2:**
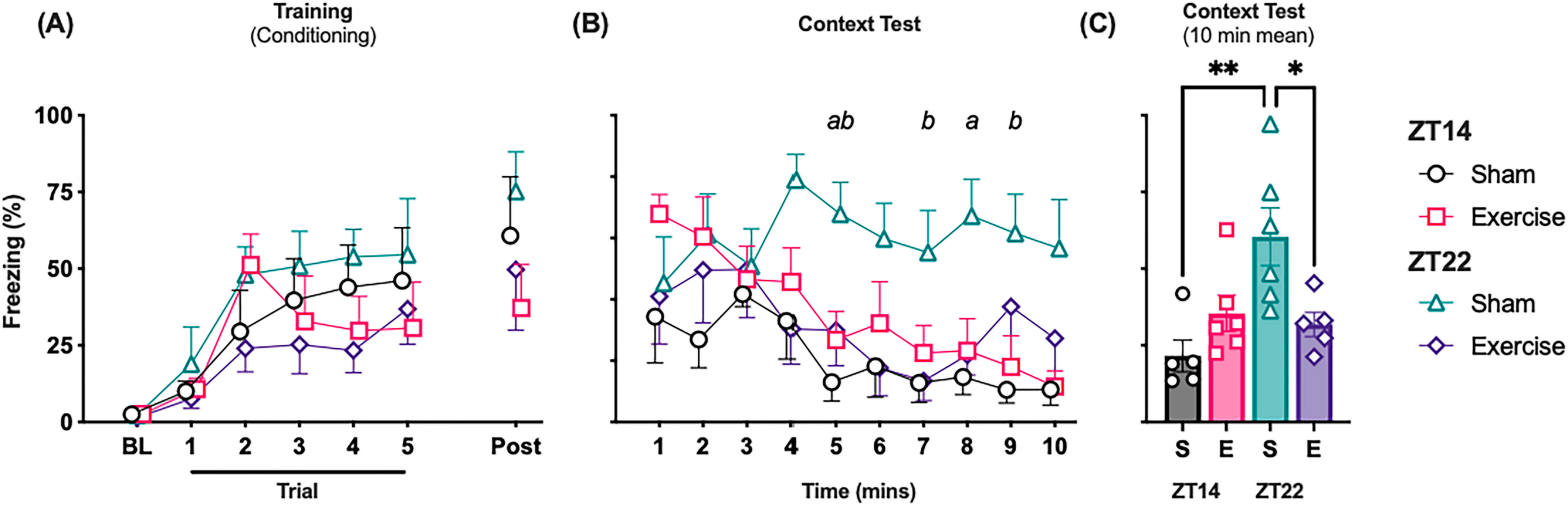
Effect of exercise timing on rats’ acquisition and retrieval fear. **(A)** Mean freezing percentage during the conditioning (Training) session. Freezing was quantified before the first trial (baseline, BL) and during the 1 min after each trial. **(B)** Mean percentage of freezing to context over 10-min test. **(C**) A total of 10-min mean freezing percentage during context fear test. All data are reported as mean ± SEM (*N* = 5–6). Significant differences identified by the mixed model are denoted as(a) ZT22 sham vs. ZT22 Exercise and (b) ZT22 Sham vs. ZT14 Sham. Significant differences between groups following one-way ANOVA are indicated by *P < 0.05 and **P < 0.01. ^a^Significantly different from Exercise at ZT22. ^b^Significantly different to Sham at ZT14.

Before the first foot shock (BL), there was no fear behavior exhibited by any animal (Figure 2A). With each successive foot shock, freezing behavior increased with a significant main effect of time (*F*_(2_._560_, _12_._80)_ = 13.47, *P* = 0.004, η^2^p = 0.73), with no difference between groups (*F*_(2_._389_, _11_._95)_ = 1.571, *P* = 0.24, η^2^p = 0.27), indicating all animals acquired the fear similarly. When freezing behavior was examined across the 10-min context test (Figure 2B), animals exhibited robust freezing in the initial minutes, reflecting retrieval of contextual fear memory and no significant difference in freezing between groups. The mixed-effects model revealed a significant main effect of group (*F*_(1_._91_, _9_._57)_ = 6.15, *P* = 0.02, η^2^p ≈ 0.39), but no significant effect of time (*F*_(2_._91_, _14_._54)_ = 3.16, *P* = 0.06) or time × group interaction (*P* = 0.23). *Post hoc* comparisons indicated that ZT22 sham rats exhibited significantly greater freezing than ZT14 sham rats during the middle and later portions of the test (minutes 5–9, *P* < 0.05). ZT22 exercised rats froze less than their sham counterparts at several time points (minutes 5 and 8, *P* < 0.05), whereas ZT14 exercise had no impact. When data were averaged across the full 10-min session (Figure 2C), there was a significant main effect for time of day (*F*_(1_, _18)_ = 6.681, *P* < 0.02, η^2^p = 0.27) and time-of-day × exercise interaction (*F*_(1_, _18)_ = 9.743, *P* < 0.01, η^2^p = 0.35) for mean freezing during the 10 min context test (Figure 2C). Freezing behavior was significantly increased in ZT22 sham rats compared to ZT14 sham rats (*P* < 0.001) whilst there was no time-of-day effect in exercise rats (*P* = 0.71). Freezing behavior was significantly reduced in ZT22 exercised rats compared to their un-exercised counterparts (*P* < 0.01), with no impact of ZT14 exercise (*P* = 0.17). Together, these findings demonstrate that sham-exercise control at ZT22 expressed greater contextual fear, whereas exercise intervention at ZT22 abolished this time-of-day effect by reducing freezing.

### Neurotransmitter and corticosterone concentrations

**FIGURE 3:**
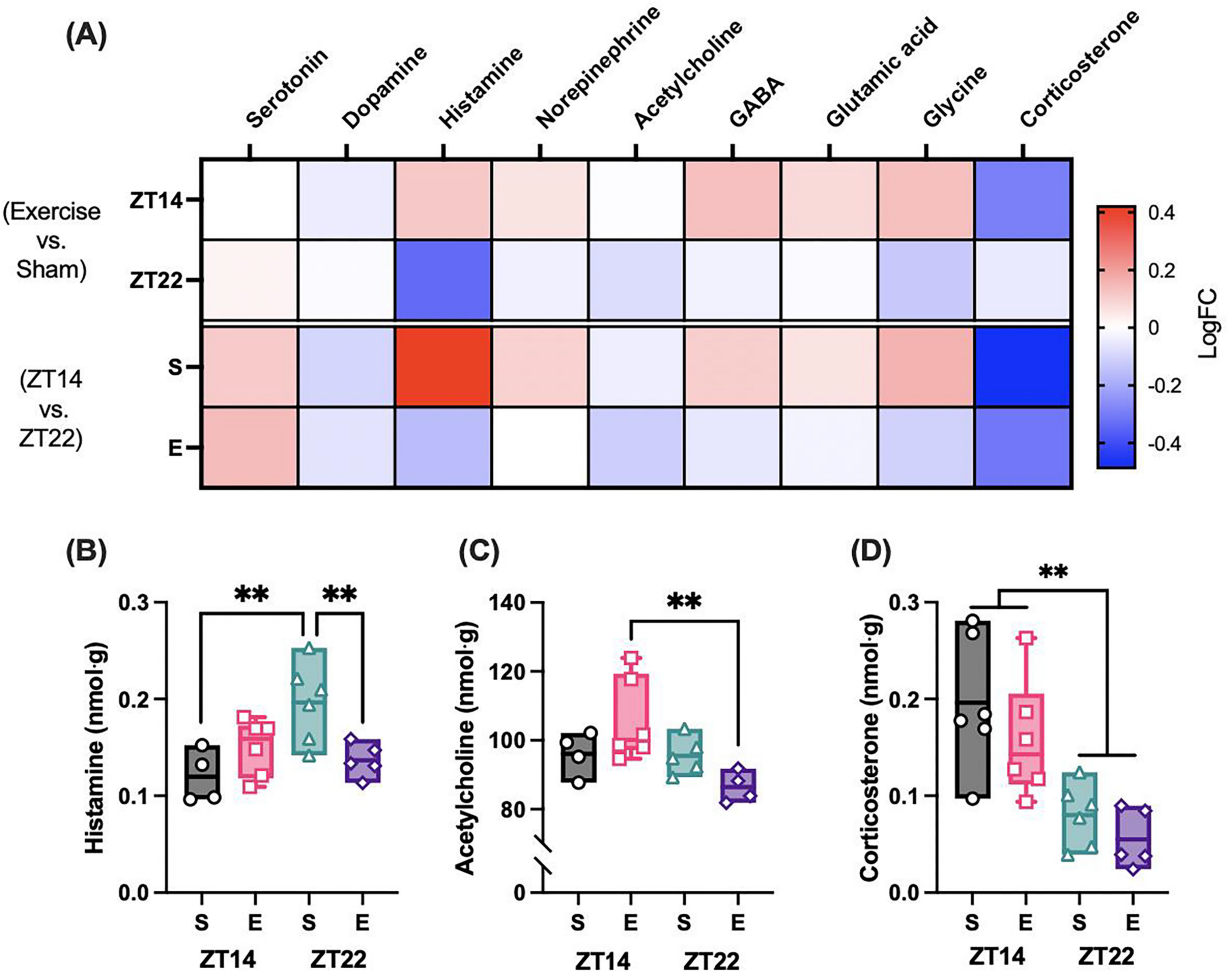
Relative hippocampal neurotransmitter and corticosterone concentrations. **(A)** Representative Heatmap of log fold change (LogFC) in neurotransmitter and corticosterone concentrations between exercise and control animals at each time point (ZT14 and ZT22). **(B–D)** Boxplots representing neurotransmitters **(B)** histamine, **(C)** acetylcholine, and **(D)** corticosterone with significant main effects. Significant differences between groups following one-way ANOVA are indicated by **P < 0.01 (*N* = 5–6). S, Sham-exercise group; E, Exercise group.

To determine whether hippocampal neurotransmitter concentrations were influenced by exercise timing, we performed mixed-effects analyses for each metabolite (Figure 3A and Supplementary Table 1). Of the nine neurotransmitters assessed, three showed significant time-of-day × exercise interactions: histamine (*F*_(1_,_7)_ = 23.09, *P* = 0.002), acetylcholine (*F*_(1_,_15)_ = 5.99, *P* = 0.027), and γ-aminobutyric acid (GABA) (*F*_(1_,_8)_ = 10.62, *P* = 0.012). In contrast, no main or interaction effects of exercise were observed for serotonin, dopamine, norepinephrine, glutamate, or glycine (all *P* > 0.1). *Post hoc* comparisons revealed distinct transmitter-specific patterns. Histamine concentrations exhibited a strong timing-dependent effect in sham animals, with higher levels in the ZT14 sham group than the ZT22 sham group (*P* = 0.001; Figure 3B). Exercise abolished this time-of-day variation, and ZT22 exercised rats displayed significantly lower histamine than ZT22 sham controls (*P* = 0.005; Figure 3B). For acetylcholine, a time-of-day effect emerged only under exercise conditions, with higher concentrations at the ZT14 group compared to the ZT22 group (*P* = 0.002; Figure 3C). GABA concentrations were higher in exercised compared to sham animals at ZT14 (*P* = 0.005), and sham animals again showed a time-of-day effect with elevated levels in the ZT22 group relative to the ZT14 group (*P* = 0.009). In addition to neurotransmitters, corticosterone levels were robustly regulated by treatment timing (*F*_(1_,_9)_ = 28.10, *P* = 0.0005), with higher concentrations in the ZT14 group compared to the ZT22 group in both sham (*P* = 0.0028) and exercised (*P* = 0.0073) animals (Figure 3D). Of note, the reduction in hippocampal corticosterone levels in ZT22 control rats, despite increased freezing behavior, may reflect a maladaptive response to chronic stress exposure, potentially associated with blunted glucocorticoid responsiveness or chronic impaired HPA axis feedback regulation (Monari et al., 2024).

### Neurotransmitter and behavioral correlations

**FIGURE 4:**
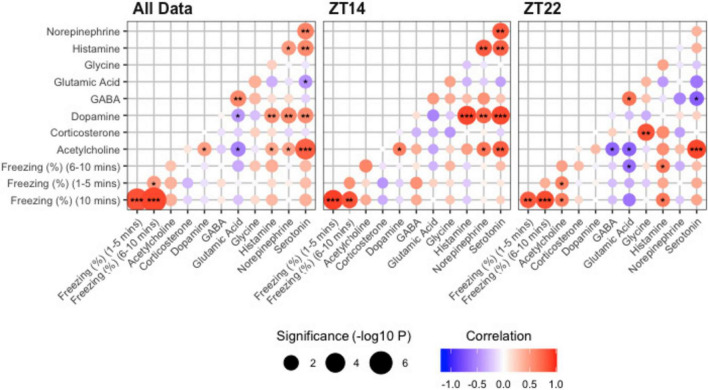
Correlation matrices (Pearson’s *r*^2^) stratified for All Data, ZT14, and ZT22. Positive and negatively correlated variables are represented by red and blue, respectively. Significantly correlated variables are annotated with *, **, *** representing *P* < 0.05, *P* < 0.01, *P* < 0.001. GABA, gamma- aminobutyric acid.

We assessed pairwise Pearson correlations among neurotransmitters, corticosterone, and freezing behavior and controlled the false-discovery rate (FDR; Benjamini–Hochberg) (Figure 4). As an internal check, freezing measures were strongly inter-correlated across the full dataset (e.g., 6–10 vs. 10 min: *r* = 0.88, *q* = 3.7 × 10^–^6), and within each timepoint (ZT14: 1–5 vs. 10 min *r* = 0.92, *q* = 4.8 × 10^–3^; ZT22: 6–10 vs. 10 min *r* = 0.87, *q* = 0.018). Across all animals pooled, no neurotransmitter–freezing associations survived FDR correction (all *q* ≥ 0.12). Nevertheless, several transmitter–transmitter relationships did. Serotonin correlated positively with acetylcholine (*r* = 0.77, *q* = 5.3 × 10^–^4), serotonin with norepinephrine (*r* = 0.61, *q* = 0.045), norepinephrine with dopamine (*r* = 0.57, *q* = 0.050), and glutamic acid with GABA (*r* = 0.58, *q* = 0.050), indicating coordinated transmitter pools at baseline.

Time-stratified analyses revealed phase-specific structure rather than direct behavior–neurotransmitter couplings. In the ZT14 group, transmitter correlations were prominent: dopamine correlated with serotonin (*r* = 0.88, *q* = 8.9 × 10^–3^), histamine (*r* = 0.89, *q* = 8.0 × 10^–3^), and norepinephrine (*r* = 0.76, *q* = 0.048); serotonin correlated with histamine (*r* = 0.77, *q* = 0.046) and norepinephrine (*r* = 0.80, *q* = 0.036); and serotonin also correlated with acetylcholine (*r* = 0.78, *q* = 0.042). No neurotransmitter–freezing associations in the ZT14 group met the FDR threshold (all *q* ≥ 0.28). In the ZT22 group, serotonin remained positively associated with acetylcholine (*r* = 0.89, *q* = 0.016). In addition, glycine correlated positively with corticosterone (*r* = 0.83, *q* = 0.035). Other nominal ZT22 trends, such as positive associations of histamine or acetylcholine with freezing and negative associations of glutamic acid with freezing, did not survive FDR correction (all *q* ≥ 0.19).

Together with the mixed-model results, these analyses indicate that exercise timing reshapes transmitter coordination in a circadian-phase-dependent manner. In contrast, direct neurotransmitter–behavior correlations are weak after multiple-testing control. This pattern is consistent with exercise altering the network organization of hippocampal transmitters (e.g., serotonergic–cholinergic and catecholaminergic clusters), rather than producing a single linear driver of freezing behavior.

## Discussion

Recent advances in chronobiology highlight that the timing of lifestyle behaviors, such as food intake and physical activity, can serve as a potent strategy for disease prevention and health optimization (Asher and Sassone-Corsi, 2015; Bennett and Sato, 2023). Building on the previous finding that acute exercise elicits time-of-day-dependent neurotransmitter responses in the hypothalamus (Sato et al., 2022), the present study investigated the long-term effects of chronically timed exercise on fear memory and hippocampal neurotransmitter profiles in rats, demonstrating that the timing of chronic exercise influences contextual fear expression in a circadian-phase-dependent manner. Sedentary rats at ZT22 expressed greater freezing during retrieval than sedentary rats at ZT14, whereas timed exercise intervention at ZT22 abolished this time-of-day difference. These findings identify a vulnerability to aversive memory expression that is buffered by exercise, if timed correctly.

These findings extend prior work showing that both physiological responses to exercise (Gabriel et al., 2021; Kemler et al., 2020; Sato et al., 2019, 2022; Savikj et al., 2019, 2022) and learning/memory processes (Maren and Holmes, 2016) are gated by circadian phase. A latest study further highlights exercise timing as a modifiable factor influencing lipid metabolism in adipose tissue (Kutsenko et al., 2025). Specifically, increased lipid synthesis was observed in adipose tissue of rats subjected late active-phase (ZT23) exercise intervention. Although the effects of chronic exercise on brain lipid metabolism remain unclear, these findings raise the possibility that late active-phase exercise may enhance systemic lipid synthesis, including in the hippocampus, thereby influencing neurotransmitter dynamics through lipid-mediated synaptic vesicle trafficking. Accumulating evidence demonstrates that exercise exerts anxiolytic effects and enhances hippocampal plasticity (Anderson and Shivakumar, 2013; Bartholomew et al., 2005; Broman-Fulks et al., 2004; Byrne and Byrne, 1993; Fulk et al., 2004; Gomes da Silva et al., 2012; Salim et al., 2010; Wang and Wang, 2016). Still, most behavioral testing in rodents has been conducted during the inactive phase, potentially confounding results with fatigue or sleep pressure (Armstrong, 1980; Baruch et al., 2004; Burghardt et al., 2006; Greenwood et al., 2009; Van Hoomissen et al., 2004). Hopkins and Bucci (2010) highlighted that interpreting exercise effects on fear conditioning depends on the timing of behavioral testing relative to circadian phase. In the present study, we aligned exercise interventions with the animals’ nocturnal activity profile (exercise at ZT14 or ZT22; retrieval at ZT18) and observed a selective reduction of freezing after exercise at ZT22. Thus, rather than broadly suppressing fear, exercise appears to normalize a phase-specific elevation in freezing during retrieval.

Mechanistically, the transmitter data suggest reorganization of neuro-modulatory tone rather than a single linear driver of behavior. Histamine showed a strong exercise × time interaction: sham animals at ZT14 exhibited lower histamine than sham animals at ZT22, whereas exercise intervention at ZT22 reduced histamine relative to sham animals and flattened this time-dependent difference. Histamine regulates arousal, attention, and hippocampal synaptic plasticity through H1/H2 (excitatory) and H3 (autoreceptor) pathways (Brown et al., 2001; Haas and Panula, 2003; Panula et al., 1989), and has been implicated in the consolidation of fear memory and anxiety (da Silva et al., 2006; Passani et al., 2007; Zarrindast et al., 2002). A decrease in histamine following exercise when baseline levels are otherwise high (ZT22) could help stabilize retrieval processes. Importantly, direct transmitter–freezing correlations did not survive false-discovery control. This pattern suggests that exercise reshapes the network organization of hippocampal transmitters (e.g., serotonergic-cholinergic and catecholaminergic clusters), instead of modulating freezing behavior via a single linear pathway.

Exercise also enhanced cholinergic rhythmicity (higher acetylcholine levels in ZT14 exercised rats than ZT22 exercised rats) and altered GABA levels (higher in ZT14 exercise rats compared to ZT14 sham rats), suggesting a shift in the excitation–inhibition balance that may favor more selective contextual retrieval. Cholinergic signaling supports context discrimination and pattern separation, whereas GABAergic tone shapes network excitability (Ballinger et al., 2016). Their coordinated adjustment could reduce over-generalized freezing without accelerating extinction. This view is consistent with our correlation matrices, which revealed serotonergic–cholinergic and catecholaminergic clusters across phases, rather than strong univariate transmitter–behavior couplings.

The neurophysiological benefits of exercise on the hippocampus may also extend to improved episodic memory (Aghjayan et al., 2022), which deteriorates during normal and pathological aging (e.g., Alzheimer’s Disease) (Caselli et al., 2009; Hedden and Gabrieli, 2004). Moreover, executive functions and memory are improved following exercise (Barha et al., 2017; Sanders et al., 2019), suggesting exercise-specific neuroplastic adaptations in cortical regions such as the prefrontal cortex (Colcombe et al., 2004, 2006; Friedman and Robbins, 2022). Improvements in memory and executive processes following exercise have significant implications for healthy aging (Boa Sorte Silva et al., 2024), and future studies should endeavor to understand the impact of exercise timing on the neuroprotective benefits of exercise.

Limitations include the use of male Long-Evans rats only; thus, potential sex differences in behavioral and neuroendocrine responses to timed exercise remain unknown. Notably, prior studies have reported sex-dependent effects of exercise on body composition in rats (Kutsenko et al., 2021). Treadmill exercise, even with sham handling, may introduce mild stress distinct from voluntary wheel running. We sampled a single post-test timepoint in the hippocampus; we did not measure in-task transmitter dynamics, receptor expression, or cell-type specificity. Behavioral assays were restricted to contextual freezing at ZT18; we did not probe cue-specific fear, extinction retention, or anxiety-like behaviors outside conditioning. Finally, while several exercise × time interactions were robust, the study was underpowered for fine-grained network analyses.

In summary, these findings suggest that exercise, if timed appropriately, buffers contextual fear while reorganizing hippocampal transmitter concentrations, particularly histaminergic, cholinergic, and GABAergic tone. The increasing global burden of mental disorders represents a significant and growing public health and social challenge. If conserved across species, chronobiology-based exercise may represent a simple, scalable intervention to modulate aversive memory expression and improve mental wellbeing in humans.

## Data Availability

The raw data supporting the conclusions of this article will be made available by the authors, without undue reservation.
