# Supplementary material for: Timing of exercise differentially modulates fear memory and hippocampal neurotransmitters in male rats
Source: Front Neurosci. 2026 May 1;20:1824029. doi: 10.3389/fnins.2026.1824029 (PMC13176273; doi:10.3389/fnins.2026.1824029)
Supplement: Supplementary file 1 [file Table_1.docx]

|  | Adjusted *P* Value | | | *Q* Value | | |
| --- | --- | --- | --- | --- | --- | --- |
| Neurotransmitter | Exercise | Time | E x T | Exercise | Time | E x T |
| Serotonin | 0.1684 | 0.8291 | 0.7565 | 0.6008 | 0.8291 | 0.9997 |
| Dopamine | 0.3004 | 0.7914 | 0.9997 | 0.6008 | 0.8291 | 0.9997 |
| Histamine | 0.2475 | **0.0158** | **0.002** | 0.6008 | 0.0808 | **0.016** |
| Norepinephrine | 0.9511 | 0.3312 | 0.4043 | 0.9511 | 0.52992 | 0.64688 |
| Acetylcholine | 0.9437 | **0.0202** | **0.0272** | 0.9511 | 0.0808 | 0.07253 |
| GABA | 0.1121 | 0.1596 | **0.0116** | 0.6008 | 0.4256 | **0.0464** |
| Glutamate | 0.3866 | 0.7791 | 0.9554 | 0.606 | 0.8291 | 0.999 |
| Glycine | 0.4545 | 0.3142 | 0.1994 | 0.606 | 0.52992 | 0.3988 |

# **Table 1 -** Adjusted P values and false discovery rate–corrected q values for the effects of exercise, time, and their interaction (Exercise × Time) on hippocampal neurotransmitter concentrations. Significant effects after FDR correction are highlighted in **bold.**
